# Supplementary material for: FunduSegmenter: Leveraging the RETFound Foundation Model for Joint Optic Disc and Optic Cup Segmentation in Retinal Fundus Images
Source: Transl Vis Sci Technol. 2026 May 19;15(5):14. doi: 10.1167/tvst.15.5.14 (PMC13206755; doi:10.1167/tvst.15.5.14)
Supplement: Supplement 1 [file tvst-15-5-14_s001.pdf]

FunduSegmenter: Leveraging the RETFound Foundation Model for Joint Optic Disc and Optic Cup Segmentation in Retinal Fundus Images

Supplementary Figures

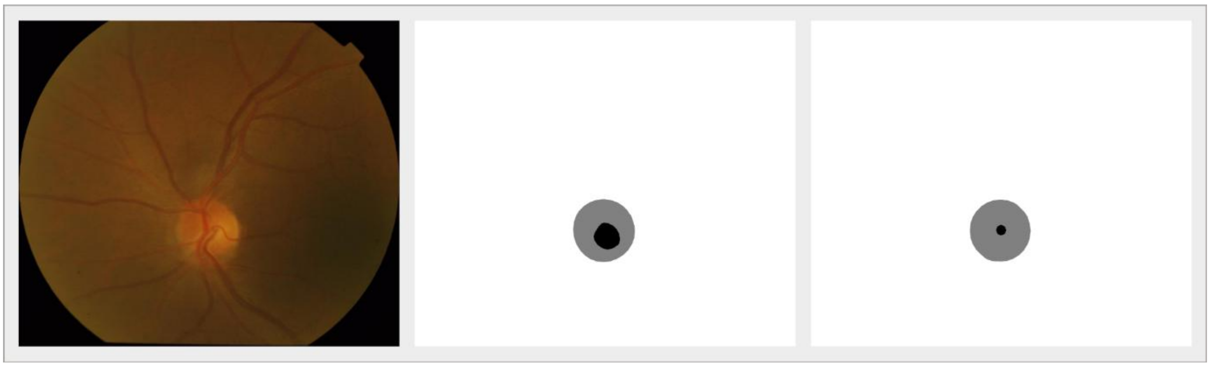

Figure S1. Drishti-GS segmentation mask example (drishtiGS_096). Left: Image. Middle: segmentation mask produced by the agreement from three experts (our way). Right: segmentation mask produced by the agreement from all experts (DoFE's way).

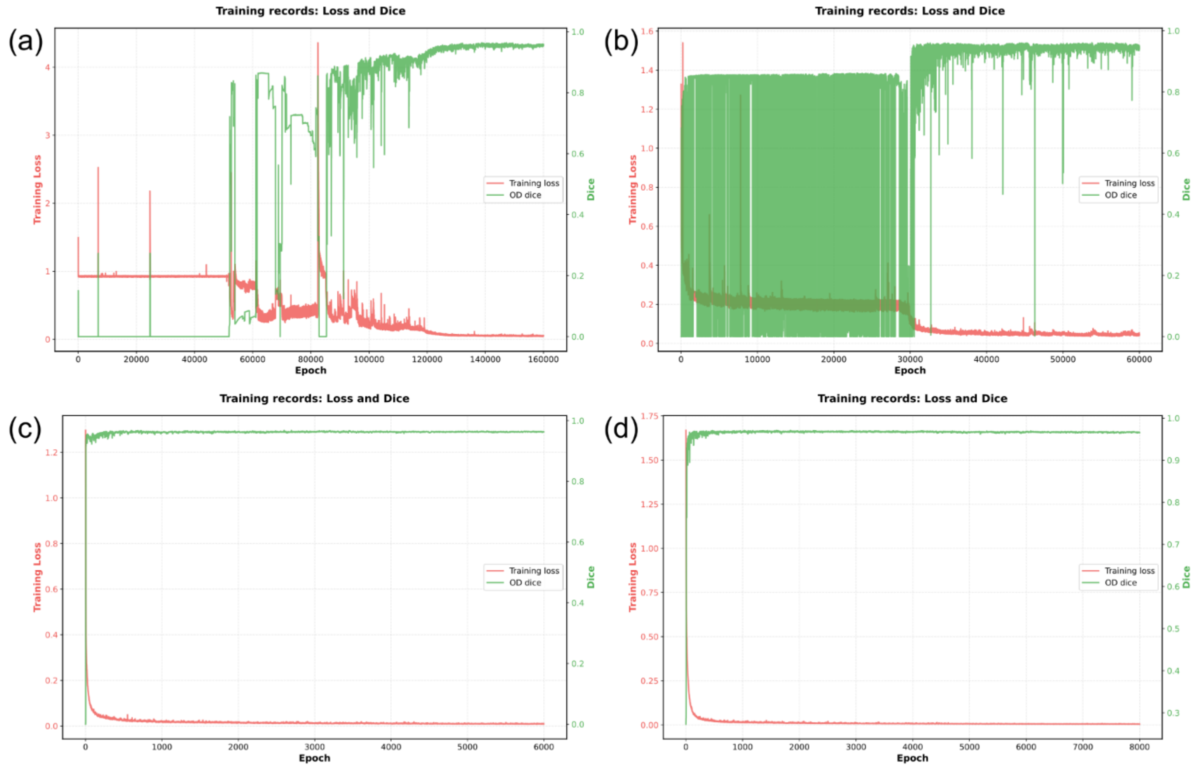

Figure S2. Training progression of model architectures with key proposed modules on IDRiD. (a) RETFound + Segmenter. (b) Pre-adapter + (a) + Post-adapter. (c) Skip connections with CBAM + (b). (d) FunduSegmenter ((c) + ViT block adapter).

Supplementary Tables

Methods	*Aug*	*Best step*	*Datasets*				*Average*
			Target (Source: GoDARTS)				
			IDRiD	Drishti-GS	RIM-ONE-r3	REFUGE	
			OD	OD	OD	OD	OD
nnU-Net	-	-	9.88 [7.70, 12.24]	35.40 [31.85, 39.06]	52.47 [47.93, 56.82]	87.19 [86.14, 88.19]	46.24
DUNet	None	80,070	93.16 [91.15, 94.77]	82.18 [76.63, 87.00]	59.85 [51.48, 67.54]	90.98 [90.43, 91.50]	81.54
	Spatial	13,480	95.09 [93.97, 96.06]	84.11 [79.49, 88.28]	66.08 [58.18, 73.09]	90.95 [90.40, 91.46]	84.06
	Designed	3,595	94.12 [92.40, 95.47]	91.72 [89.44, 93.41]	73.84 [67.34, 79.56]	90.79 [90.24, 91.31]	87.62
TransUNet	None	1,810	88.09 [85.98, 90.01]	81.78 [79.28, 84.13]	90.02 [88.53, 91.34]	**92.04 [91.48, 92.57]**	87.98
	Spatial	3,110	91.37 [90.06, 92.62]	83.65 [81.24, 85.91]	**91.00 [90.01, 91.93]**	91.45 [90.88, 91.98]	89.37
	Designed	920	88.59 [85.33, 91.32]	82.17 [79.30, 84.77]	89.22 [88.11, 90.29]	91.21 [90.62, 91.78]	87.80
Ours	None	155	92.12 [91.24, 92.93]	94.95 [94.18, 95.60]	88.95 [88.06, 89.78]	91.79 [91.45, 92.12]	91.95
	Spatial	620	92.99 [92.20, 93.74]	95.30 [94.43, 96.07]	89.90 [89.12, 90.66]	90.63 [90.17, 91.07]	92.21
	Designed	910	**95.41 [94.91, 95.92]**	**95.54 [94.83, 96.18]**	88.73 [87.93, 89.51]	91.31 [90.85, 91.74]	**92.75**

Table S1. Results of our method compared with state-of-the-art baselines on the external verification task (Source: GoDARTS). All figures are DSC (%) with 95% CI. Average OD denotes the simple average of OD DSC.

Methods	*Aug*	*Best step*	*Datasets*				*Average*
			Target (Source: IDRiD)				
			GoDARTS	Drishti-GS	RIM-ONE-r3	REFUGE	
			OD	OD	OD	OD	OD
nnU-Net	-	-	**93.11 [91.63, 94.56]**	92.97 [89.01, 95.57]	79.07 [72.95, 84.14]	63.21 [59.33, 66.99]	82.09
DUNet	None	1,775	78.11 [69.77, 85.01]	69.21 [60.87, 76.90]	40.47 [32.39, 48.26]	77.04 [75.82, 78.23]	66.21
	Spatial	16,756	82.01 [75.48, 87.78]	76.48 [69.16, 83.10]	47.96 [39.42, 56.15]	91.24 [90.53, 91.89]	74.42
	Designed	1,046	89.35 [87.00, 91.65]	93.25 [91.92, 94.52]	76.03 [71.82, 79.88]	84.49 [83.55, 85.42]	85.78
TransUNet	None	6,220	71.96 [60.56, 82.20]	95.87 [95.09, 96.52]	81.93 [78.70, 84.73]	70.25 [68.87, 71.65]	80.00
	Spatial	897	78.29 [69.58, 85.97]	95.13 [94.17, 96.01]	80.84 [77.62, 83.67]	86.49 [85.50, 87.46]	85.19
	Designed	3,550	87.55 [84.55, 90.04]	94.66 [93.83, 95.46]	83.02 [80.28, 85.27]	86.75 [85.68, 87.80]	88.00
Ours	None	93	89.97 [87.84, 91.94]	96.40 [95.78, 96.90]	**89.61 [87.74, 91.06]**	90.79 [90.32, 91.24]	91.69
	Spatial	5,242	91.25 [89.59, 92.85]	**97.09 [96.79, 97.37]**	88.17 [87.11, 89.22]	**93.74 [93.33, 94.13]**	**92.56**
	Designed	1,845	90.85 [88.97, 92.54]	97.05 [96.75, 97.34]	86.91 [85.96, 87.81]	91.90 [91.32, 92.46]	91.68

Table S2. Results of our method compared with state-of-the-art baselines on the external verification task (Source: IDRiD). All figures are DSC (%) with 95% CI. Average OD denotes the simple average of OD DSC.

Methods	*Aug*	*Best step*	*Datasets*						*Average*		
			Target (Source: Drishti-GS)								
			GoDARTS	IDRiD	RIM-ONE-r3		REFUGE				
			OD	OD	OD	OC	OD	OC	OD	OC	All
nnU-Net	-	-	69.76 [63.82, 75.81]	66.63 [62.48, 70.59]	84.10 [81.59, 86.32]	60.19 [54.81, 65.33]	88.16 [87.07, 89.23]	**76.03 [74.51, 77.55]**	77.16	68.11	74.15
DUNet	None	8,668	66.18 [57.40, 74.67]	68.83 [61.98, 75.35]	74.90 [69.57, 79.42]	54.76 [49.43, 60.04]	28.39 [27.43, 29.41]	11.52 [10.73, 12.38]	59.58	33.14	50.76
	Spatial	10,764	65.26 [56.86, 73.82]	78.45 [70.90, 85.43]	76.20 [70.79, 80.95]	56.34 [50.70, 61.86]	32.18 [30.74, 33.76]	12.05 [10.96, 13.20]	63.02	34.20	53.41
	Designed	7,703	80.30 [75.68, 84.85]	94.03 [92.68, 95.28]	85.54 [83.73, 87.17]	**69.49 [64.77, 73.86]**	67.39 [66.15, 68.61]	43.43 [41.56, 45.34]	81.82	56.46	73.36
TransUNet	None	413	69.06 [57.62, 79.80]	93.47 [91.33, 95.09]	77.21 [74.28, 79.90]	53.60 [48.19, 59.03]	66.58 [65.40, 67.74]	52.79 [51.03, 54.54]	76.58	53.20	68.79
	Spatial	3,979	81.21 [75.76, 85.97]	94.27 [92.25, 95.85]	82.60 [80.67, 84.32]	63.47 [58.42, 68.29]	66.39 [65.40, 67.37]	43.51 [41.97, 45.02]	81.12	53.49	71.91
	Designed	4,740	79.82 [72.57, 86.35]	93.36 [90.56, 95.62]	80.69 [78.31, 82.85]	58.85 [53.68, 63.81]	60.52 [59.30, 61.74]	34.07 [32.70, 35.44]	78.60	46.46	67.89
Ours	None	107	88.14 [85.19, 90.69]	90.81 [85.57, 95.10]	80.23 [76.45, 83.33]	55.10 [49.43, 60.54]	85.72 [84.81, 86.59]	59.05 [57.42, 60.63]	86.23	57.08	76.51
	Spatial	5,181	83.44 [78.09, 88.43]	95.78 [94.37, 96.71]	**85.76 [84.45, 87.07]**	67.74 [63.25, 72.01]	**90.07 [89.46, 90.65]**	65.04 [63.75, 66.38]	88.76	66.39	81.31
	Designed	1,250	**88.96 [86.23, 91.34]**	**96.13 [95.44, 96.68]**	85.59 [84.54, 86.61]	65.61 [61.15, 69.90]	88.63 [88.07, 89.17]	73.61 [72.55, 74.67]	**89.83**	**69.61**	**83.09**

Table S3. Results of our method compared with state-of-the-art baselines on the external verification task (Source: Drishti-GS). All figures are DSC (%) with 95% CI. Average OD, OC and 'All' denote the simple average of OD, OC and all DSC.

Methods	*Aug*	*Best step*	*Datasets*						*Average*		
			Target (Source: RIM-ONE-r3)								
			GoDARTS	IDRiD	Drishti-GS		REFUGE				
			OD	OD	OD	OC	OD	OC	OD	OC	All
nnU-Net	-	-	55.01 [50.71, 58.99]	54.50 [51.30, 57.66]	82.90 [80.19, 85.44]	58.45 [54.00, 62.77]	51.40 [50.26, 52.53]	28.70 [27.34, 30.14]	60.95	43.58	55.16
DUNet	None	1,862	46.93 [39.52, 54.43]	60.30 [55.30, 65.51]	81.59 [78.39, 84.41]	60.44 [56.08, 64.50]	36.04 [35.13, 37.01]	29.59 [28.32, 30.86]	56.22	45.02	52.48
	Spatial	2,448	50.75 [43.40, 58.49]	65.93 [60.32, 71.13]	78.72 [74.76, 82.29]	60.79 [56.93, 64.51]	46.92 [45.69, 48.20]	52.32 [50.43, 54.26]	60.58	56.56	59.24
	Designed	2,600	68.63 [64.00, 73.21]	68.36 [61.72, 75.07]	89.37 [87.77, 90.73]	71.69 [68.72, 74.47]	71.38 [70.40, 72.36]	64.92 [63.30, 66.53]	74.44	68.31	72.39
TransUNet	None	1,292	68.07 [62.80, 72.78]	76.83 [72.52, 80.71]	88.79 [87.96, 89.54]	**72.15 [69.58, 74.66]**	75.74 [74.66, 76.78]	48.47 [46.19, 50.81]	77.36	60.31	71.68
	Spatial	478	71.36 [66.42, 75.84]	86.21 [84.91, 87.43]	90.55 [89.89, 91.15]	64.82 [61.36, 68.17]	87.05 [86.67, 87.43]	71.21 [69.77, 72.63]	83.79	68.02	78.53
	Designed	622	73.84 [69.89, 77.41]	88.62 [87.56, 89.58]	91.24 [90.58, 91.87]	69.70 [66.65, 72.64]	87.40 [87.00, 87.79]	66.38 [64.43, 68.25]	85.28	68.04	79.53
Ours	None	398	73.12 [69.29, 76.58]	84.04 [82.77, 85.27]	91.39 [90.75, 91.97]	61.30 [57.78, 64.85]	88.02 [87.63, 88.40]	73.29 [72.20, 74.39]	84.14	67.30	78.53
	Spatial	3,968	69.63 [65.39, 73.66]	85.37 [84.57, 86.18]	89.68 [89.07, 90.28]	59.09 [55.42, 62.71]	**89.39 [89.10, 89.68]**	**79.33 [78.39, 80.25]**	83.52	**69.21**	78.75
	Designed	836	**79.19 [76.49, 81.71]**	**88.91 [88.17, 89.68]**	**92.05 [91.60, 92.49]**	61.12 [57.90, 64.33]	88.64 [88.24, 89.03]	74.69 [73.38, 75.99]	**87.20**	67.91	**80.77**

Table S4. Results of our method compared with state-of-the-art baselines on the external verification task (Source: RIM-ONE-r3). All figures are DSC (%) with 95% CI. Average OD, OC and 'All' denote the simple average of OD, OC and all DSC.

Methods	*Aug*	*Best step*	*Datasets*						*Average*		
			Target (Source: REFUGE)								
			GoDARTS	IDRiD	Drishti-GS		RIM-ONE-r3				
			OD	OD	OD	OC	OD	OC	OD	OC	All
nnU-Net	-	-	66.21 [52.91, 78.34]	43.61 [31.03, 56.31]	95.88 [95.12, 96.54]	**83.40 [80.86, 85.73]**	87.50 [86.09, 88.60]	79.40 [75.34, 82.85]	73.30	**81.40**	76.00
DUNet	None	54,948	50.84 [39.39, 61.82]	71.79 [64.33, 78.49]	78.92 [72.93, 84.01]	56.53 [49.57, 62.81]	53.24 [44.96, 61.17]	40.24 [32.02, 48.46]	63.70	48.39	58.59
	Spatial	4,476	52.94 [39.20, 66.41]	78.37 [71.43, 84.60]	86.88 [82.02, 90.83]	58.56 [52.12, 64.54]	66.55 [60.12, 72.34]	50.39 [41.88, 58.60]	71.19	54.48	65.62
	Designed	1,452	60.79 [49.07, 72.33]	79.91 [74.69, 84.61]	92.84 [89.82, 94.89]	73.49 [69.55, 77.02]	72.67 [66.46, 78.12]	66.31 [59.47, 72.46]	76.55	69.90	74.34
TransUNet	None	672	61.36 [51.61, 70.91]	89.33 [85.50, 92.72]	91.64 [89.73, 93.35]	53.45 [49.66, 57.15]	85.93 [84.32, 87.26]	74.14 [70.07, 77.69]	82.07	63.80	75.98
	Spatial	2,772	73.56 [66.85, 79.67]	94.66 [93.42, 95.70]	93.41 [91.32, 95.17]	62.45 [59.17, 65.75]	87.58 [86.69, 88.46]	80.87 [76.52, 84.50]	87.30	71.66	82.09
	Designed	888	71.05 [65.75, 76.52]	90.83 [86.75, 94.11]	94.13 [92.48, 95.51]	73.72 [70.51, 76.73]	**88.44 [87.66, 89.21]**	78.10 [74.93, 81.02]	86.11	75.91	82.71
Ours	None	1,668	73.93 [67.47, 80.22]	93.57 [92.19, 94.79]	96.25 [95.68, 96.75]	67.29 [63.00, 71.14]	85.95 [84.60, 87.15]	79.09 [75.81, 82.15]	87.43	73.19	82.68
	Spatial	396	**78.98 [73.54, 84.20]**	93.71 [92.11, 95.05]	96.06 [95.69, 96.43]	76.34 [73.45, 79.04]	84.81 [83.67, 85.89]	**81.81 [78.31, 84.94]**	88.39	79.08	85.29
	Designed	3,684	77.41 [72.00, 82.48]	**94.91 [93.98, 95.78]**	**96.67 [96.29, 97.02]**	76.29 [73.70, 78.75]	87.70 [86.81, 88.55]	80.79 [77.57, 83.74]	**89.17**	78.54	**85.63**

Table S5. Results of our method compared with state-of-the-art baselines on the external verification task (Source: REFUGE). All figures are DSC (%) with 95% CI. Average OD, OC and 'All' denote the simple average of OD, OC and all DSC.

No.	*Model*	*Size*	*Best step*	*Datasets*					*Average*
				Source	Target				
				IDRiD	GoDARTS	Drishti-GS	RIM-ONE-r3	REFUGE	
				OD	OD	OD	OD	OD	All
17	RET + Seg(2)	224	147,237	96.32	90.08	96.19	85.15	79.24	89.40
18	Pre-A + Best + Post-A	256	53,788	95.86	89.14	95.94	88.24	79.41	89.72
19	Best + Skip	256	2,600	96.87	90.98	96.51	87.57	91.02	92.59
20	**FunduSegmenter**	256	1,845	96.88	90.85	97.05	86.91	91.90	**92.72**

Table S6. Results of ablation study Group 2 experiments. All figures are DSC (%). Average 'All' denotes the simple average of all DSC. Acronyms used in this table denote the same meaning in Table 6.

Methods	*Pre-processing*	*Best step*	*Datasets*					
			Source	Target				
			GoDARTS	IDRiD	Drishti-GS	RIM-ONE-r3	REFUGE	*Average*
			OD	OD	OD	OD	OD	All
DUNet	OD center crop	3,595	75.35	94.12	91.72	73.84	90.79	87.62
	Original	21,520	95.88	97.69	89.82	18.58	86.91	73.25
TransUNet	OD center crop	920	65.05	88.59	82.17	89.22	91.21	87.80
	Original	15,800	95.82	97.63	90.64	62.35	90.71	85.33
Ours	OD center crop	205	78.99	95.41	95.54	88.73	91.31	92.75
	Original	910	95.20	96.66	92.88	91.56	91.70	93.20

Table S7. Results of ablation study Group 3 experiments. All figures are DSC (%). Average 'All' denotes the simple average of external experiment DSC.
